# Supplementary material for: Variations in the Abortive HIV-1 RNA Hairpin Do Not Impede Viral Sensing and Innate Immune Responses
Source: Pathogens. 2021 Jul 15;10(7):897. doi: 10.3390/pathogens10070897 (PMC8308900; doi:10.3390/pathogens10070897)
Supplement: Supplementary file 1 [file pathogens-10-00897-s001.zip › pathogens-1303803-supplementary.pdf]

Table S1. Quantitative Real-Time PCR primers to analyze mRNA expression

**Gene product**	Forward primer	**Reverse primer**
GAPDH	CCATGTTCGTCATGGGTGTG	GGTGCTAAGCAGTTGGTGGTG
IFNB	ACAGACTTACAGGTTACCTCCGAAAC	CATCTGCTGGTTGAAGAATGCTT
APOBEC3G	TTGAGCCTTGGAATAATCTGCC	TCGAGTGTCTGAGAATCTCCCC
IL27A	GCTTTGCGGAATCTCACCTG	TGAAGCGTGGTGGAGATGAAG
ISG15	TTTGCCAGTACAGGAGCTTGTG	GGGTGATCTGCGCCTTCA
IRF3	AAGGAGGCGTGTTTGACCTG	CATAGCGTGGTGAGCGT
IRF7	GCTCCCCACGCTATACCATCTAC	GCCAGGGTTCCAGCTTCAC
